# Action of Medicines on the Urinary Secretions

**Published:** 1864-09

**Authors:** 


ART. V.—*Action of Medicines on the Urinary Secretions.*

A chapter on the variations of the urinary secretion by the action of medicine in the recent work on this subject, by Dr. Edward Parkes, (London, 1861,) affords a curious illustration of the facts of modern physiological science. What service this diffluent excrete has rendered to physic in the days of blue-bottle mystery; what lives have been saved by it in spite of the labeled elixir that required such noted shaking before the kidney received its contents to cast them to the dogs, let the succeeding epitome of our author's inquiries in this direction explain.

Antimony emerges, in part or altogether, by the kidneys. Its mode of combination is uncertain, and the rate at which it passes out is also unknown.

When the salts of arsenic, lead and copper are taken, the metal passes off partly by the urine. Salts of iron, especially, augment largely the iron of the urine. Lead, although it may pass off in large quantities, does so much more slowly. Copper passes off by urine as well as in the fæces.

Mercury passes off largely by the kidneys; but it is yet not proved that, under the influence of this drug, temporary albuminuria is a result.

Silver, in part, issues by the kidneys. The combination in the urine is not known.

Zinc, according to Sclossberger, may be detected in the urine after administration of the oxide. But the quantity is small; and, in the opinion of Heller, nil. Zinc is excreted in by far the largest quantity by the fæces.

Sulphur passes off by the urine in part, at least, as sulphuric acid, and in part uncombined.

Sulphuret of potassium, when not taken in too large a quantity, is oxydized, and appears as sulphate of potash in the urine.

Iodine appears very rapidly in the urine after being taken, and is in combination probably with sodium.

Sulphuric, nitric, phosphoric and hydrochloric acids, all emerge by the kidney, and augment the acidity of the urine. This effect increases with the increase of the dose. The acids take alkali from the system; phosphoric acid seems to take potash; sulphuric acid, soda and potash. The combinations of hydrochloric and nitric acids are unknown. According to Eylandt, all these acids pass out in twenty-four hours

Oxalic, tartaric, citric and acetic acids are converted in the system into carbonates. Citric and acetic acids appear to be entirely destroyed; oxalic and tartaric in most part. All increase the acidity of the urine, chiefly by the formation of carbonic acid, which in part appears in the urine.

Benzoic acid, and all substances containing benzoyl, or oil of bitter almonds, cause the appearance of hippuric acid. There is now no doubt that this occurs from the combination of benzoic acid and glycin. This combination probably takes place in the hepatic cells. Cinnamic acid, and balsam of tolu, which contains it, also produce hippuric acid. Nitro benzoic acid produces nitro hippuric acid.

Gallic acid passes unchanged into the urine; Dr. Parkes thinks that it does not influence the elimination of urea or of uric acid. Pyrogallic acid passes unchanged.

Tannic acid passes off by the urine in the forms of gallic and pyrogallic acids, and perhaps of a saccharine body.

Uric acid and the urates of potash or ammonia cause a considerable increase of urea, the acid being converted almost, if not entirely, into carbonic acid and urea.

Hippuric acid passes unchanged in the urine.

Lactic acid is decomposed, perhaps even in the stomach. Whether or not it is transferred into the urine is uncertain.

Liquor potassæ passes out of the system by the urine probably in six hours, with increase of secreted water. The condition of the urea is doubtful; the uric acid is apparently unaffected; the pigment seems not to be increased; the chloride of sodium is sometimes increased; the sulphuric acid is increased; the phosphoric acid is increased, the alkali apparently passing off in combination with these acids; the free acidity of the urine is lessened.

Ammonia appears to be transformed into a salt in the body, and to pass off as such, principally as chloride of ammonium. An hypothesis has been raised that ammonia is oxydized in the system, and appears as nitric acid in the urine. Dr. Parkes records that the point is undecided, but rather favors the hypothesis. The evidence on which the speculation rests is innocent, but naturally not robust.

The carbonates of soda are rapidly excreted by the kidneys, and render the urine alkaline more or less quickly. The soda is probably excreted in the form of carbonate or bicarbonate. Nitrate of soda passes out by the kidneys; phosphate of soda and phosphoric acid have actions which bear a certain resemblance to each other. Phosphoric acid, when taken pure, passes out combined. The alkali it carries out is almost entirely potash; the acid does not take lime or magnesia. As in passing out of the system it removes potash, it might be presumed, says Dr. Parkes, that the administration of phosphoric acid would tend to carry away so much base as to "de-alkalize" the body; but this presumption is incorrect, for Boeker has discovered the singular fact that the acid ($PO_5$) diminishes very greatly the elimination of chloride of sodium—to such an extent, indeed, that, in spite of the increased excretion of potash, the total amount of alkali leaving the system is diminished; the same effect is produced by the phosphate of soda. Phosphate of soda also causes a diminution in the amount of urea and of water; but phosphoric acid has no such effect. The acid increases the amount of iron; the salt does not. Phosphate of soda is not a diuretic, but the reverse.

Carbonate of potassa renders the urine alkaline. It is not yet known whether or not the potash leaves the body in any other form than that in which it entered. Although this salt makes the urine alkaline, it augments in some diseased conditions the acidity of the urine; for the acidity of the urine is found, after the salt has been left off, to be greatly increased.

Nitrate of potash is very rapidly excreted by the urine, the acidity of which it does not destroy. According to Biegel, the nitrate does not increase metamorphosis in health, nor yet act as a diuretic. Dr. Parkes does not altogether endorse this opinion.

Acetate of potash, which, as the late Dr. Golding Bird taught, was supposed to increase the excretion of urinary solids, seems, by later observations made on healthy men, to lessen such excretion. Yet certainly the acetate of potash will sometimes increase the elimination of water. The acetate is invariably transformed into the carbonate in the system, and renders the urine alkaline.

The citrate and the tartrate of potash produce, like the acetate, alkalinity of urine from the formation of carbonate; the amount of free carbonic acid is also greatly increased.

When iodide of potassium is taken by the mouth, iodine can speedily be detected in the urine. The salt is eventually almost entirely excreted by the kidneys. Possibly also, but the point is not proved, this salt increases the amount of urine. Iodide of potassium eliminates mercury from the body.

Chlorate of potash, in large doses, increases the acidity of the urine, and is said by Isambert to cause a more free deposit of urates and pigment.

Sulpho-cyanide of potassium passes out unchanged, and ferro-cyanide of potassium becomes ferri-cyanide.

The salts of ammonia pass out of the urine unchanged, and Boeker states that the chloride increases all the constiuents, except the uric acid, which it slightly diminishes. The tartrate of ammonia does not increase the acidity of the urine.

The influence of phosphate and of lactate of lime on the urine is not correctly known.

Chloride of calcium causes an increase in the amount of lime; the magnesia is slightly increased, and the acidity is lessened.

Quinine and cinchonine pass off by the urine in part. In healthy persons, quinine appears in the course of two to five hours. In some diseases the excretion of the alkaloid is much delayed; this obtains in intermittents. Some quinine is always destroyed in the body, and Ranke observed that the amount of uric acid was materially lessened during the administration of the disulphate of quinine. The urea remains unaffected.

Morphia, in great part, passes unchanged into the urine. This drug, as well as opium, produces rather variable effects in regard to the amount of water; the solids appear to be always decreased.

Belladonna increases, rather than diminishes, the urinary water, the urea, and the extractives. Atropine passes off, in part at least, by the urine, from which it can be recovered.

Digitalin, when taken in doses of seven one-hundredths part of a grain a day, cannot be detected in the urine. Under the influence of digitalis, the water would appear to be increased, the fixed salts lessened, and the organic solids unaltered. The action of the drug differs in health from that which follows its administration in disease, a difference perhaps explained by its action on the heart and great vessels.

Strychnine passes off in part by the urine, though whether all that is taken can be recovered is not known. The effects on the constituents is also unknown. Veratrin and nicotin are not proved to be diuretics.

Colchicin and colchicum affect the quantity of urine variably. The urea and the uric acid, the volatile salt, extractives, and earthy phosphates are all lessened, according to some observers; but the facts are not complete as a whole. Dr. Parkes sums up by saying that colchicum rather lessens than increases the water—perhaps from its action on the bowels; it produces rather a lessening than an increase of urea and uric acid, and its effect on the other constituents is unknown.

The action of squills and juniper is as yet not absolutely determined.

Salicin passes off in part by the urine in the undecomposed state, and is in part decomposed; it would seem to take oxygen from the body both by direct metamorphosis, and also by supplying sugar, which is oxydized and turned into carbonic acid and water.

When amygdalin is taken into the body in large quantities, formic acid is detected in the urine. Mannite taken by the stomach is passed in small part—one-tenth—by the urine; the remainder is decomposed either in the stomach or intestines, being converted into lactic acid. Glycyrrhizin goes off entirely by stool, and does not appear in the urine. Amanita muscaria passes off unchanged in the urine, communicating to that fluid intoxicating properties.

Copaiva consists of three parts—an etheral oil, a resinous acid, and a neutral resin. According to Weikart, the ethereal oil does not appear in the urine, but the resinous acid (copaivic,) which forms 40 per cent. of the balsam, is eliminated to a large amount. Weikart suggests that the action of this acid on the mucous membrane is attributable to its effects on the fatty acids contained in the pus-cells formed by the inflamed membrane; the cells become shrivelled, and their nutrition and growth arrested.

Creasote may, perhaps, give rise to carbolic acid, as a dark colour of the urine is sometimes seen after its use. Turpentine gives a violet color to the urine; its effects on the urinary constituents are not known.

Cod-liver oil, according to Beneke, invariably lessens the free acidity of the urine; an action ascribed by him to the lessened disintegration of the nitrogenous tissues.

Cantharides, in medicinal doses, sometimes increases, sometimes decreases, the amount of urine; its further effects are as yet undiscovered.

